# Effects of C/EBPα overexpression on alveolar epithelial type II cell proliferation, apoptosis and surfactant protein-C expression after exposure to hyperoxia

**DOI:** 10.1186/s12890-019-0911-x

**Published:** 2019-08-06

**Authors:** Hongyan Lu, Xiaoqing Chen, Yanmin Lu, Haitao Zhu, Wei Tang, Qiuxia Wang

**Affiliations:** 1 Department of Pediatrics, Affiliated Hospital of Jiangsu University, Zhenjiang, 212000 China; 2 Department of Pediatrics, the First Affiliated Hospital of Nanjing Medical University, Nanjing, Jiangsu China

**Keywords:** AEC II cells, Apoptosis, C/EBPα, Hyperoxia, Proliferation, Surfactant protein-C

## Abstract

**Background:**

This study aims to investigate the effects of CCAAT/enhancer binding protein alpha (C/EBPα) overexpression on cell proliferation, apoptosis and surfactant protein-C(SP-C) in alveolar epithelial type II (AEC II) cells after exposure to hyperoxia.

**Methods:**

pcDNA3.1(+)-C/EBPα plasmid or air-empty vector were transfected into AEC II cells with or without hyperoxia. AEC II cells were divided into air group, air+pcDNA3.1-C/EBPα group, air-empty vector group, hyperoxia group, hyperoxia+pcDNA3.1-C/EBPα group, and hyperoxia-empty vector group. Cell proliferation was analyzed using Cell Counting Kit-8. The mRNA level and protein expression were measured using PCR and Western blot techniques, respectively. The cell cycle and apoptosis were analyzed using flow cytometry.

**Results:**

After 48 h of post-transfection, significantly higher protein expression of C/EBPα was observed in the C/EBPα transfection group with or without hyperoxia compared to the others (*P* < 0.05). Compared to the air group, hyperoxia decreased cell proliferation, increased apoptosis, decreased SP-C expression, decreased percentage of cells in G1 phase, and increased percentage of cells in the S and G2 phases (*P* < 0.05); however, reversed by C/EBPα transfection (*P* < 0.05). No significant changes were observed in cell proliferation, SP-C expression, and apoptosis rates in the C/EBPα transfection group as compared to the controls air-empty vector group.

**Conclusion:**

C/EBPα overexpression significantly upregulates the expression of SP-C, promotes cell proliferation, and inhibits apoptosis in AEC II cells after exposure to hyperoxia. Hence, this data suggests that C/EBPα overexpression may reverse the damage and exert a protective role in hyperoxia-induced lung injury.

**Electronic supplementary material:**

The online version of this article (10.1186/s12890-019-0911-x) contains supplementary material, which is available to authorized users.

## Introduction

Hyperoxia-induced lung injury (HILI) is one of the major causes of death and morbidity in premature infants, especially those with extremely low birth weight [1, 2]. Nevertheless, the exact pathogenesis of HILI is yet unclear. Some investigators speculate that oxidative stress plays a critical role in the occurrence and development of HILI [3]. Furthermore, the inhalation of high concentrations of oxygen can lead to the secretion of alveolar surfactant proteins, inhibition of cell proliferation and differentiation, and increased apoptosis of alveolar epithelial type II (AEC II) cells [4]. CCAAT/enhancer binding protein alpha (C/EBPα) is the first member of the C/EBP family, a basic region-leucine zipper (bZIP) protein family [5]. The expression and regulation of *C/EBPα* gene has been reported in the pathogenesis of several common diseases, including chronic obstructive pulmonary disease, asthma, lung cancer, acute myelogenous leukemia, and renal diseases [6–14]. In recent years, studies have found that as a key transcription factor regulating cell proliferation and differentiation, C/EBPα is essential for the lung development as well as injury [15]. Berg et al. found that C/EBPα is extensively expressed in AEC II, airway epithelial cells, and lung macrophages during the vesicular and alveolar phases of lung development; the abnormal expression of C/EBPα in lung cells affects the lung development [16]. In fetal rat which lacks C/EBPα gene, pulmonary surfactant protein synthesis is decreased, AEC II differentiation is inhibited, and lung maturation disorder and alveolar process are interrupted, thus, indicating that C/EBPα may be a vital transcription factor for the maturation of fetal lung [17]. Although C/EBPα plays a major role in lung development, researchers demonstrated that the internal environmental homeostasis of adult rat lung does not require the expression of C/EBPα gene under unstressed conditions. In spite of depletion of the C/EBPα gene in the adult rat lungs, the morphology and function of the lungs remain normal. However, *C/EBPα* gene-deficient mature rats are sensitive to hyperoxia, following which, severe lung inflammation and decreased expression of surfactant protein-B (SP-B) are observed in mice, thereby indicating that C/EBPα exerts a protective role in hyperoxia-induced lung injury [18, 19]. In a previous study, we demonstrated that in the early stage of hyperoxia exposure, C/EBPα promotes the secretion of pulmonary surfactant protein and participates in the protective regulation of the body. However, over the course of hyperoxia exposure, C/EBPα loses compensatory protective effects [20]. At present, whether the overexpression of C/EBPα after hyperoxia can reverse the function of AEC II cells, including proliferation and differentiation, remains unclear. Herein, we hypothesized that C/EBPα plays a major role in lung protection from respiratory epithelial cell injury. Therefore, we investigated the effects of C/EBPα overexpression on AEC II cell proliferation, apoptosis, and surfactant protein-C (SP-C) after exposure to hyperoxia and lay a foundation to study the pathogenesis and the prevention of hyperoxia-induced lung injury.

## Materials and methods

### Reagents

All the materials and reagents were as follows: human primary type II alveolar epithelial cells (AEC II cells); cat. no. HUM-iCELL-a002Human donor information: Male, 52 years old, Chinese, Lung cancer patient, Non-malignant tissue samples were obtained from pneumectomy specimens; purchased from iCell Bioscience, Inc., Shanghai, China); RPMI1640 (GE Healthcare HyClone life Sciences, USA); OPTI-MEM (Gibco, Thermo Fisher Scientific Inc., USA); fetal bovine serum (FBS; Wisent Inc., China); pcDNA3.1(+)-C/EBPα, negative control pcDNA and primers (Sangon Biotech Co., Ltd., China); trypsin, lipofectamine 2000 and TRIzol (Invitrogen, Thermo Fisher, USA); sodium dodecy1 sulfate-polyacrylamide gel electrophoresis (SDS-PAGE), polyvinylidene difluoride (PVDF) membranes and RNase enzyme (CWbiotech, China); antibodies against C/EBPα and SP-C (Santa Cruz Biotechnology Inc., USA); RNA LA PCR~ (TM) and SYBR Premix Ex Taq™ (TaKaRa Biomedical Technology, China); β-actin and mouse anti-rabbit HRP-conjugated antibodies (Cell Signaling Technology Inc., USA); rabbit anti-sheep HRP-conjugated antibody (FCMAC Biomedical Technology Ltd., China); Cell Counting Kit-8 (CCK-8; Biosharp, Hefei, China); Propidium Iodide (PI) Staining Kit (Keygen Biotech, China); FITC AnnexinV/PI Kit (BD Company, USA); and CYS-1 digital oxygen meter (JDxuelian Factory, China).

### Cell culture and grouping

The cells were cultured in RPMI-1640 medium supplemented with 10% FBS and 100 U/mL penicillin-streptomycin in a humidified atmosphere containing 5% CO_2_ / 95% O2 air at 37 °Celsius. After reaching 80–90% confluency, the cells were divided into air group, air-empty vector group, air+pcDNA3.1-C/EBPα group, hyperoxia group, hyperoxia+pcDNA3.1-C/EBPα group and hyperoxia-empty vector group.

### Cell transient transfection and exposure to hyperoxia

24 h before transfection, the cell culture medium was replaced with fresh medium. After reaching 50% confluency, the transfection was performed using Lipofectamine 2000 reagent, according to the manufacturer’s instructions. The OPTI-MEM medium was used during transfection. The transfected cells were cultured in serum-free culture medium, and fresh medium added after 48 h. Subsequently, the cells were treated with air or hyperoxia. The air groups were maintained in an environment of 5% CO_2_ in air at 37 °C, while the hyperoxia cells were exposed to 5% CO_2_ in 95% O_2_, for 10 min, followed by culturing in a CO_2_ incubator at 37 °C. The oxygen concentration of the hyperoxia cells was tested using oxygen monitor when the gases were replaced every day; the samples in which the oxygen concentration was < 90%, were discarded.

### Real-time polymerase chain reaction (RT-PCR)

After exposure to hyperoxia or air for 48 h, the cells were washed with cold phosphate-buffered saline (PBS) at pH 7.2. Total RNA was extracted using TRIzol, and the concentration and purity were evaluated using a UV spectrophotometer (Thermo Fisher Scientific, USA); the RNA samples were stored at − 80 °C. Next, the cDNA was synthesized according to the instructions of the reverse transcription kit (TaKaRa Biomedical Technology, China). The corresponding primer sequences are shown in Table 1. The specificity of the PCR reactions was evaluated by melting curve analysis. The threshold cycle (Ct) values were set, and relative expression of the target gene was calculated based on the 2^-ΔΔCt^ method [ΔCt = Ct (target gene) - Ct (internal control), and ΔΔCt = ΔCt (experiment group) - ΔCt (control group)].

**Table 1:** Sequences of primers

Name	Primer sequence	Product
C/EBPα	Forward: 5′-GGTGGACAAGAACAGCAACG-3′	124 bp
	Reverse: 5′-GGTCAGCTCCAGCACCTTCT-3′	
SP-C	Forward: 5′- TTACCACTGCCACCTTCTCC-3’	139 bp
	Reverse: 5′-TCAAGACTGGGGATGCTCTC-3’	
β-actin	Forward: 5′-GCAGAAGGAGATTACTGCCCT-3’	136 bp
	Reverse: 5′-GCTGATCCACATCTGCTGGAA-3’	

### Western blot

Cells were harvested after exposure to hyperoxia or air for 48 h. All the cells were washed with ice-cold PBS and solubilized with the radioimmunoprecipitation assay (RIPA) buffer containing protease inhibitor Phenylmethanesulfonyl fluoride (PMSF) (Sigma-Aldrich; Merck KGaA, Darmstadt, Germany) for 30 min at 4 °C with agitation. The extract was centrifuged at 12,000×*g*, 4 °Celsius for 15 min, and the supernatant collected. Following normalization of protein concentrations of the samples using the BCA kit (Beyotime; Shanghai, China), 20 μg of the cell lysates were resolved by 12% SDS-PAGE and transferred to polyvinylidene difluoride (PVDF) membranes (CWbiotech, China). Subsequently, the membranes were blocked using 5% milk-Tris-buffered saline (TBS) containing 0.1% Tween-20 (TBST) at 37 °C for 1 h, followed by incubation with primary antibodies, including sheep monoclonal anti-C/EBPα (1:200), rabbit monoclonal anti-SP-C (1:500) and rabbit monoclonal anti-β-actin (1:1000) at 4 °C overnight. Then, the membranes were washed with TBST and incubated with secondary antibodies for 1 h at 37 °C, including rabbit anti-sheep horseradish peroxidase (HRP)-conjugated (1:1000 anti-C/EBPα) and mouse anti-rabbit HRP conjugated (1:1000 anti-SP-C and anti-β-actin). β-actin was used as a loading control. The immunoreactive bands were visualized by chemiluminescence (ProteinSimple, San Jose, CA, USA). The protein expressions were quantified by densitometric analysis using LANE 1D software (Sage, Beijing, China).

### Cell proliferation

After exposure to hyperoxia or air for 48 h, CCK-8 was used to evaluate the cell proliferation. Cells were plated in 96-well plates (5000 cells/well) and incubated in a CO_2_ incubator at 37 °C. After 24 h, 10 μL CCK-8 was added to each well and incubated for another 2 h, following which, the absorbance was quantified at 450 nm using an automated enzyme-linked immunosorbent assay (ELISA) reader. The cell proliferation was calculated according to the following formula: *number of cells = (OD experimental group – OD blank group) / (OD control group – OD blank group)*; *cell proliferation rate = number of cells/number of cells at 0 h).* OD indicates the optical density; the OD value is directly proportional to the number of cells proliferating.

### Cell cycle analysis

Cell cycle distribution was determined by PI staining using flow cytometry. After exposure to hyperoxia or air for 48 h, cells were trypsinized, centrifuged, washed with cold PBS, and resuspended in PBS containing 70% ethanol to obtain a single-suspension (1–5 × 10^5^ cells/mL). This suspension was mixed with 500 μL PI and RNase A and incubated at 4 °C for 30 min. The cell cycles were analyzed using a flow cytometer (CANTO 10C, BD Bioscience, USA), and data analyzed by ModFit LT 4.1 software (Verity Software House Inc., Topsham, ME, USA).

### Cell apoptosis analysis

Apoptosis was determined with AnnexinV-fluorescein isothiocyanate (FITC)/PI using flow cytometer. After exposure to hyperoxia or air for 48 h, the cells were trypsinized, centrifuged, and washed with cold PBS. Then, the cells were resuspended in 100 μL binding buffer, mixed with 5 μL Annexin V + FITC and 5 μL PI, followed by incubation at room temperature for 15 min. Finally, the cells were mixed with 400 μL binding buffer. The cell apoptosis was analyzed using a flow cytometer following the manufacturer’s protocol. The apoptosis rate was analyzed using FlowJo 7.6 software (Tree Star Inc., Ashland, OR, USA).

### Statistical analysis

Statistical analyses were conducted using SPSS17.0 statistical software. Results are expressed as mean ± standard deviation. Comparison among multiple groups was performed using a one-way analysis of variance (ANOVA) and post hoc analysis between groups by SNK-q test. *P* < 0.05 was considered as statistically significant.

## Results

### pcDNA3.1(+)-C/EBPα induces increased C/EBPα protein expression

**Fig. 1:**
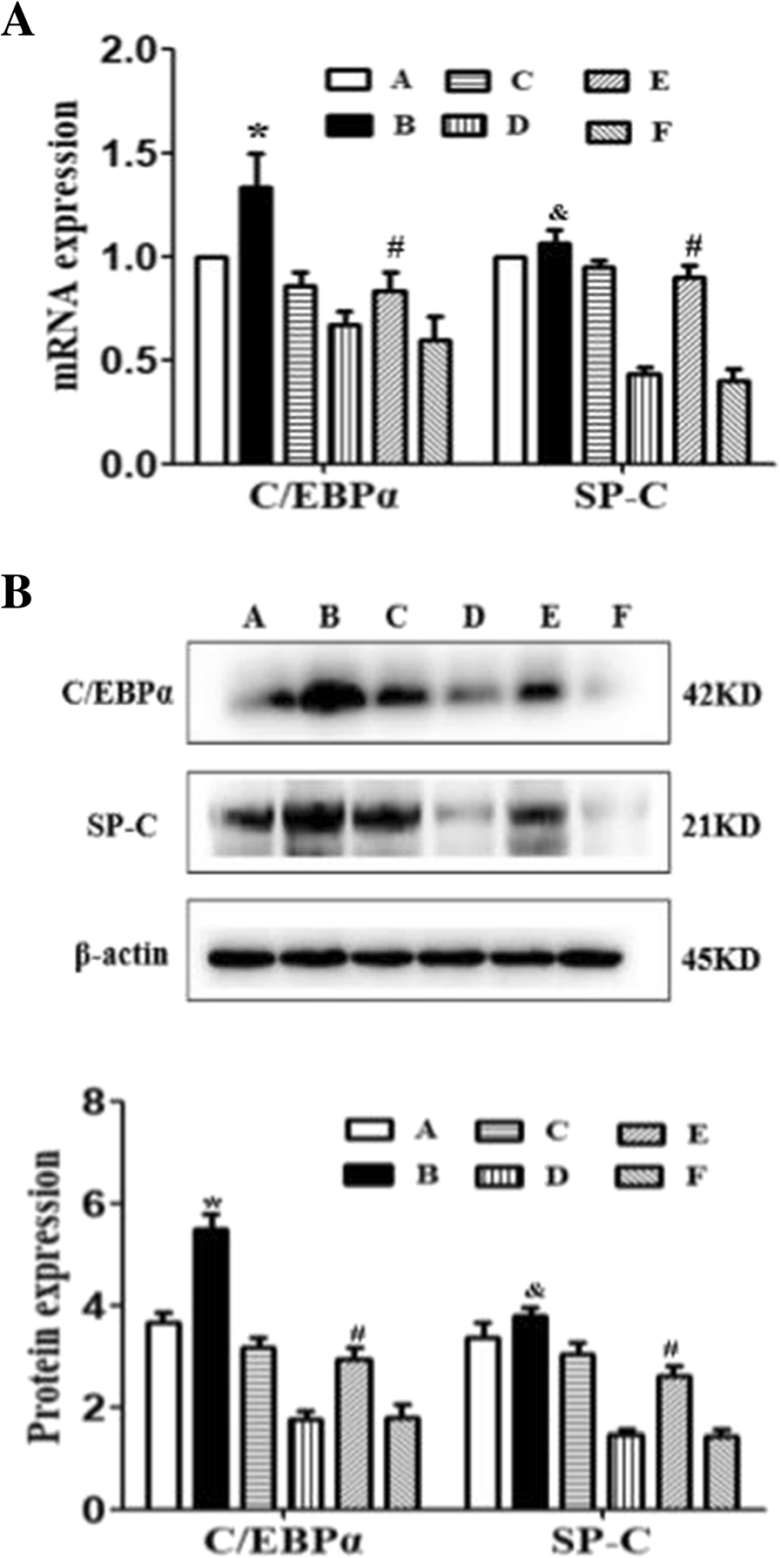
Overexpression of C/EBPα improves the secretion of SP-C in AEC II after exposure to hyperoxia . **a** RT-PCR and **b** western blot analysis of C/EBPα and SP-C mRNA and protein in the AEC II cells. β-actin was used as the loading control. Values are mean ± SD from ten independent experiments (*n* = 10); *P < 0.05 vs. control group. A: control group; B: pcDNA3.1(+)-C/EBPα group; C: pcDNA3.1(+)-empty group. D: Hyperoxia group; E: Hyperoxia+pcDNA3.1(+)- C/EBPα group; F: Hyperoxia+pcDNA3.1(+)-empty group

AEC II cells were transfected with pcDNA3.1*(+)*-C/EBPα or pcDNA3.1*(+)-*empty vector plasmid using Lipofectamine 2000 with or without hyperoxia. The total mRNA and protein was extracted 48 h post-transfection and analyzed by Western blot and PCR As shown in Fig. 1. Full-length blots were presented in Additional file 1: Figure S1.

The levels of C/EBPα mRNA and protein expression were significantly increased in C/EBPα transferred group as compared to controls (*P* < 0.05), while a similar result was obtained when comparing hyperoxia group and hyperoxia-empty vector groups with the hyperoxia+C/EBPα group (*P* < 0.05).

### Overexpression of C/EBPα improves the secretion of SP-C in AEC II after exposure to hyperoxia

Previous study showed that the expression of C/EBPα can be improved after a short period of exposure to hyperoxia, since it exerts a protective regulatory effect on the pulmonary function during the early stage of hyperoxia stress. However, the tolerance to hyperoxia injury was limited in rats. When the exposure time is prolonged, high oxygen eventually leads to severe cell injury, decreased production of C/EBPα, and rapid deterioration of the structure and function of type II AECs. Compared to the air group, the expression of C/EBPα in hyperoxia group was decreased, while the protein and mRNA expressions of SP-C in hyperoxia group and hyperoxia-empty vector groups decreased significantly (*P* < 0.05) (Fig. 1). These findings suggest that C/EBPα plays a key role in the regulation of SP-C expression after exposure to hyperoxia.

As shown in Fig. 1, the level of CEBPa mRNA and protein expression is significantly increased by transfection in hyperoxia (*P* < 0.05), while the level of expression of SP-C in the hyperoxia+pcDNA3.1-C/EBPα group was significantly higher as compared to hyperoxia and hyperoxia-empty vector groups (*P* < 0.05). However, no significant changes were observed in the SP-C expression in the C/EBPα group as compared to the air group and air-empty vector groups (*P* > 0.05). Thus, these results indicate that the overexpression of C/EBPα can improve the secretion of SP-C in AEC II cells when exposed to hyperoxia.

### Overexpression of C/EBPα promotes cell proliferation after exposure to hyperoxia

C/EBPα plays a major role in regulating the proliferation and differentiation of AECs. CCK-8 was used to evaluate the cell proliferation. As shown in Table 2 and Additional file 1: Figure S2, a significantly lower cell proliferation was detected in the hyperoxia group and hyperoxia-empty vector groups as compared to air group (*P* < 0.05). These results suggest the participation of C/EBPα in cell proliferation of AECII under hyperoxic conditions.

**Table 2:** Overexpression of C/EBPα promotes cell proliferation after exposure to hyperoxia

Group	OD value
Air (AG)	1.91 ± 0.63
Air+pcDNA3.1(+)-C/EBPα (A + C)	1.89 ± 0.52^&^
Air+ pcDNA3.1(+)-empty (A + E)	1.87 ± 0.46
Hyperoxia (HG)	0.89 ± 0.44*
Hyperoxia+pcDNA3.1(+)- C/EBPα (H + C)	2.43 ± 0.25^#^
Hyperoxia+pcDNA3.1(+)-empty (H + E)	0.76 ± 0.32*

Cell proliferation was analyzed using CCK-8 assay following transfection of C/EBPα after 48 h. Values are means ± SD from ten independent experiments (*n* = 10); *P < 0.05 vs. AG; ^#^*P* < 0.05 vs. HG or H + E; ^&^*P* > 0.05 vs. AG or A + E

In order to confirm the role of C/EBPα in cell proliferation, AECIIs were transfected with pcDNA3.1*(+)*-C/EBPα vector plasmid to assess its protective effect under hyperoxic conditions. It was observed that there was significantly higher cell proliferation in the hyperoxia+pcDNA3.1-C/EBPα group as compared to the hyperoxia group and hyperoxia-empty vector groups (*P* < 0.05). The overexpression of C/EBPα did not exhibit a significant effect on the cell proliferation of AECII as a result of air exposure. Overall, these data indicate that the overexpression of C/EBPα can promote the proliferation of AEC II cells when exposed to hyperoxia.

### Overexpression of C/EBPα regulates cell cycle distribution after hyperoxia exposure

**Fig. 2:**
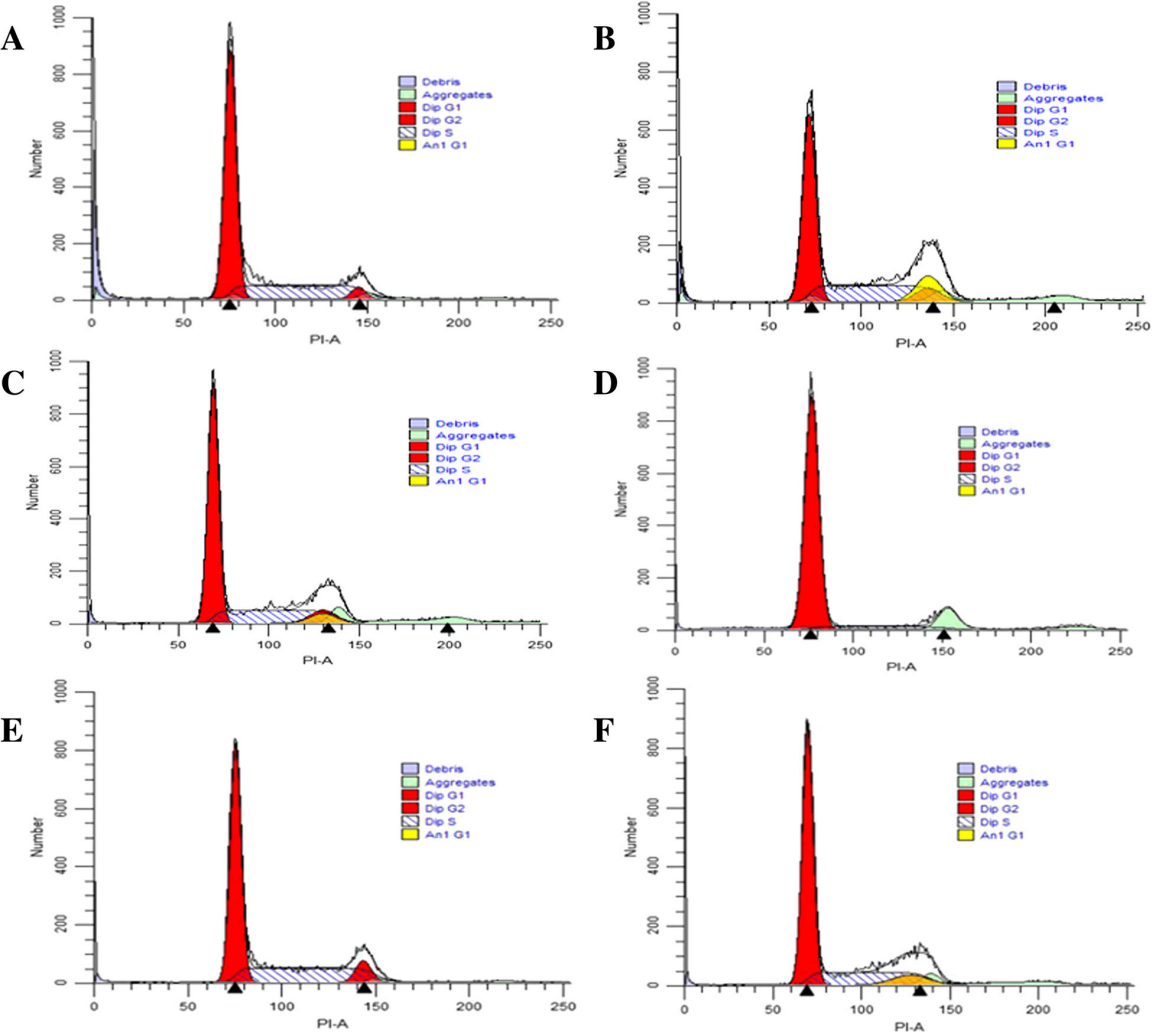
Cell cycle change in each group. *P < 0.05 vs. air group; ^#^*P* < 0.05 vs. hyperoxia group or hyperoxia-empty vector group; ^&^*P* > 0.05 vs. group or air-empty vector group. **a**: air group; **b**: C/EBPα group group; **c**: air-empty vector group; **d**: hyperoxia group; **e**: hyperoxia+pcDNA3.1-C/EBPα group; **f**: hyperoxia-empty vector group

**Fig. 3:**
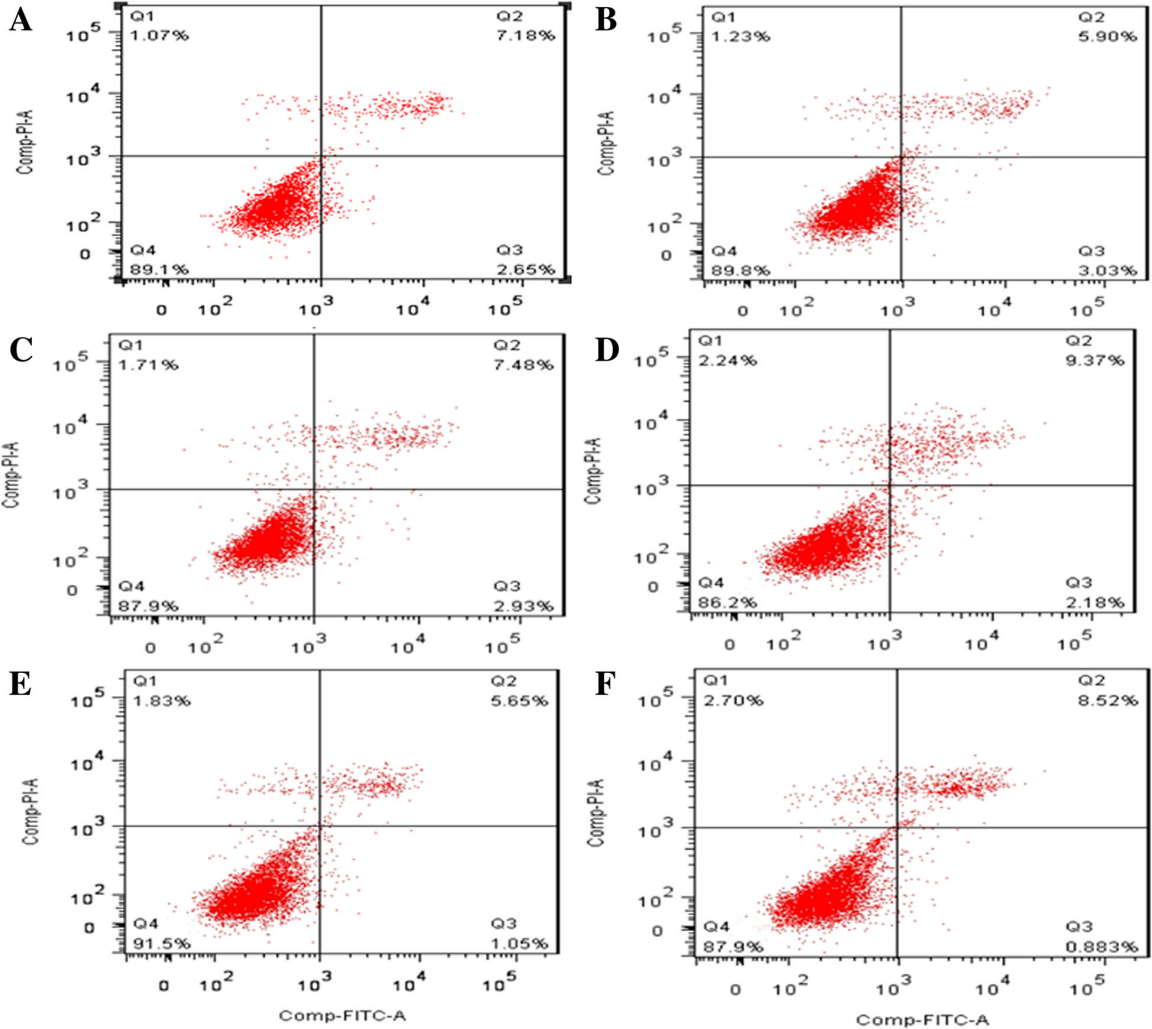
Annexin V/PI double-staining flow cytometry analysis of cell apoptosis. Q1: Necrotic Cells; Q2: Late apoptotic cells; Q3: Early apoptotic cells; Q4: Living cells. **a**: air group; **b**: C/EBPα group group; **c**: air-empty vector group; **d**: hyperoxia group; **e**: hyperoxia+pcDNA3.1-C/EBPα group; **f**: hyperoxia-empty vector group

It has been reported that C/EBPα has an important role in regulating cell cycle. We used PI staining in flow cytometry assay to detect the cell cycle distribution. As shown in Fig. 2 and Table 3, the percentage of AEC II cells in the hyperoxia group and hyperoxia-empty vector groups were increased in the G1 phase and decreased in the S and G2 phases of the cell cycle (*P* < 0.05) as compared to the air group. The part of original data for Fig. 3 were presented in Additional file 1: Figure S4. Our data indicate that C/EBPα is related to cell cycle distribution after hyperoxia exposure, and that C/EBPα is an important regulator of proliferation in AEC II.

**Table 3:** Overexpression of C/EBPα regulates cell cycle distribution after hyperoxia exposure

Cell	G1 (%)	S (%)	G2 (%)
Air (AG)	68.61 ± 2.68	28.19 ± 2.45	4.17 ± 1.87
Air+pcDNA3.1(+)- C/EBPα (A + C)	64.92 ± 3.12^&^	28.11 ± 2.53^&^	7.51 ± 2.98^&^
Air+pcDNA3.1(+)-empty (A + E)	67.26 ± 2.56	29.77 ± 1.98	6.02 ± 2.73
Hyperoxia (HG)	91.09 ± 2.41*	5.95 ± 0.98*	3.00 ± 1.61*
hyperoxia+pcDNA3.1(+)-C/EBPα (H + C)	51.15 ± 3.36^#^	37.61 ± 2.76^#^	10.29 ± 2.02^#^
Hyperoxia+pcDNA3.1(+)-empty (H + E)	92.29 ± 1.97*	6.41 ± 3.17*	2.01 ± 1.91*

Values are means ± SD from ten independent experiments (*n* = 10); *P < 0.05 vs. AG; ^#^*P* < 0.05 vs. HG or H + E; ^&^*P* > 0.05 vs. AG or A + E

To further support the speculation that overexpression of C/EBPα induces cell proliferation, we analyzed the cell cycle at 48 h after overexpression of C/EBPα. Compared with the hyperoxia group and hyperoxia-empty vector group, overexpression of C/EBPα downregulates the G1 phase, while upregulates the S and G2 phases. Conversely, the C/EBPα group and air-empty vector groups did not show any significant changes in the G1, S and G2 phases at 48 h post-transfection as compared to the air group. These results suggest that the overexpression of C/EBPα can increase the duration of S and G2 phases of the cell cycle.

### Overexpression of C/EBPα decreases cells apoptosis and necrosis after exposure to hyperoxia

To corroborate the results, we also examined the induction of apoptosis and necrosis by flow cytometry with dual staining for Annexin V and PI. Annexin-V is a cellular protein employed for the detection of apoptotic cells, while the PI dye is an intercalating agent commonly used for identifying the necrotic cells (dead cells). The apoptosis rate = early apoptosis rate (Q3) + late apoptosis rate (Q2). At 48-h post-cell transient transfection, the cell apoptosis and necrosis rates in the hyperoxia group and hyperoxia-empty vector groups were significantly increased as compared to the air group (*P* < 0.05), which suggest that C/EBPα take part in the regulation of cell apoptosis and necrosis after exposure to hyperoxia.

However, an opposite effect was observed in the hyperoxia+pcDNA3.1-C/EBPα group, wherein the cell apoptosis and necrosis were significantly decreased as compared to the hyperoxia group and hyperoxia-empty vector groups (*P* < 0.05). The significant decrease in early apoptotic cells, late apoptotic cells, and dead cells concludes that the overexpression of C/EBPα decreases the cell death when exposed to hyperoxia. In the air group, C/EBPα group, and air-empty vector groups, no significant difference was observed with respect to apoptosis and necrosis. In conclusion, our results showed that the overexpression of C/EBPα suppressed the cell apoptosis and necrosis when exposed to hyperoxia (Fig. 3, Table 4 and Additional file 1: Figure S3). The part of original data for Fig. 3 were presented in Additional file 1: Figure S5.

**Table 4:** Overexpression of C/EBPα decreases cells apoptosis and necrosis after exposure to hyperoxia

Group	Apoptosis rate
Air (AG)	9.76 ± 0.67
Air+pcDNA3.1(+)-C/EBPα (A + C)	8.97 ± 0.46^&^
Air+pcDNA3.1(+)-empty (A + E)	9.36 ± 0.39
Hyperoxia (HG)	13.01 ± 0.86*
Hyperoxia+pcDNA3.1(+)-C/EBPα (H + C)	9.51 ± 0.62^#^
Hyperoxia+pcDNA3.1(+)-empty (H + E)	13.88 ± 0.54*

Apoptosis rate of cells was analyzed by flow cytometry in each group. Values are means ± SD from ten independent experiments (*n* = 10); *P < 0.05 vs. AG; ^#^*P* < 0.05 vs. HG or H + E; ^&^*P* > 0.05 vs. AG or A + E

## Discussion

C/EBPα is a transcription factor that is crucial for lung development and the differentiation of pulmonary epithelium [21–23]. Lung epithelial cell damage occurs within 24 h post-oxygen exposure before the onset of morphological injury. In the present study, we induced AEC II injury by exposure to hyperoxia. The role and mechanisms underlying C/EBPα that influence pulmonary cytoprotection during hyperoxia injury were identified. In comparison to the air group, the expression of C/EBPα and SP-C in the hyperoxia group decreased significantly, the percentage of AEC II cells increased in G1 phase and decreased in S and G2 phases of the cell cycle, the cell proliferation decreased, and cell apoptosis increased. These results were in agreement with those described previously [23–26], thereby suggesting that high concentration of oxygen can alter the expression levels of *C/EBPα* gene in lung cells, block the G1 phase, delay the entry of cells into S phase, inhibit the synthesis of DNA, and ultimately lead to cell proliferation inhibition, increased apoptosis, and compromised lung cell function. Xu et al. [27] investigated the role of *C/EBPα* gene in the pathogenesis of lung injury by establishing a *C/EBPα* gene deficiency mouse model. The study found that mice had a decreased pulmonary compliance, while type II cells could not differentiate into type I cells after hyperoxia exposure, the expression of SP-B and SP-C decreased significantly in the bronchoalveolar lavage fluid, and several gene loci were expressed abnormally. This suggested that C/EBPα may participate in the pathogenesis of hyperoxia induced lung cell injury by affecting the secretion of surfactant protein and cell differentiation.

Chronic high oxygen exposure decreases the levels of C/EBPα and the impairment of cell proliferation and differentiation [27, 28]. Furthermore, whether the overexpression of C/EBPα can restore the lung cell function after hyperoxia exposure was investigated in this study. Briefly, we observed the effects of C/EBPα overexpression on the proliferation, apoptosis, and surfactant protein-C in AEC II cells after hyperoxia exposure. The current study transiently transfected the pcDNA3.1(+)-C/EBPα plasmid in AEC II cells; consequently, the expression of C/EBPα was significantly higher as compared to the empty plasmid group, indicating a successful transfection. Compared to the hyperoxia group, the mRNA and protein expression of SP-C and cell proliferation increased in the hyperoxia+pcDNA3.1(+)-C/EBPα group, the percentage of cells in G1 phase decreased, increased in S and G2 phases, and the apoptosis rate decreased. These findings suggested that the overexpression of C/EBPα may partially reverse the damage by hyperoxia-induced lung cell injury. Moreover, we did not find any significant changes in the SP-C expression, cell proliferation, and apoptosis rates in the C/EBPα group as compared to the air group and air-empty vector groups. Furthermore, it can be proved that C/EBPα did not play a significant role in the air group, which was in agreement with the previous study that designated a critical role of C/EBPα in postnatal pulmonary function under normal conditions and C/EBPα-mediated protection of the lung during acute lung injury induced by hyperoxia [27]. Sato et al. [29] established a mouse AEC damage model by intraperitoneal injection of naphthalene and found that C/EBPα may regulate the regeneration of bronchial epithelial cells by regulating the protease/antiprotease balance. This phenomenon suggested that C/EBPα may exert a protective role in the lung via several mechanisms. However, according to the current study, we found that overexpression of C/EBPα did not cause significant effects on cell proliferation, apoptosis and SP-C expression in AEC II with air exposure, as the AEC II used in our study was adult alveolar epithelial type II cells, suggesting that C/EBPα might not play a critical role in the function of mature alveolar epithelial cells under normal conditions.

A potential limitation of the study was liquid culture model of hyperoxia which may not be the optimum method of cell culture as compared with air-liquid interface culture which could provide a better environment to simulate the growth, differentiation and maturation of airway epithelial cells. Another issue that needs to be clarified is that the primary cells from a single donor were not representative of the general population. However, taking into account of the fact that type II alveolar epithelial cells from multiple donors would also bring more confounding factors to the experiment, we chose a single donor to ensure the reliability and stability of the experiment.

In conclusion, hyperoxia exposure can lead to decreased C/EBPα and SP-C expression, cell cycle G1 arrest, DNA synthesis inhibition, decreased cell proliferation, and increased cell apoptosis. C/EBPα overexpression has a protective role in hyperoxia-induced lung cell injury. However, the AEC II cells belong to the cell line and cannot simulate the environment of the organism completely, therefore, the protective effects of C/EBPα on hyperoxia-induced lung injury necessitates further exploration.

## Conclusions

C/EBPα overexpression significantly upregulated the expression of SP-C, promoted proliferation, inhibited apoptosis, and also increased the percentage of AEC II cells in S and G2 phases after exposure to hyperoxia. Thus, the C/EBPα overexpression may reverse the damage and exert a protective role in hyperoxia-induced lung cell injury.

## Additional file

Additional file 1: **Figure S1.** Uncropped Western blots for Fig. 1**. Figure S2.** Overexpression of C/EBPα promotes cell proliferation after exposure to hyperoxia. **Figure S3.** Overexpression of C/EBPα decreases cells apoptosis and necrosis after exposure to hyperoxia. **Figure S4.** The part of original data for Fig. 2. **Figure S5.** The part of original data for Fig. 3. (PDF 823 kb)

## Data Availability

The data and materials generated during and/or analysed during the current study are available from the corresponding author on reasonable request.

## Abbreviations

AEC II: Alveolar epithelial type II
C/EBPα: CCAAT/enhancer binding protein alpha
FBS: Fetal bovine serum
PI: Propidium iodide
RT-PCR: Real-time polymerase chain reaction
SDS-PAGE: Sodium dodecyl sulfate-polyacrylamide-gel electrophoresis
SP-C: Surfactant protein-C
