# Supplementary material for: Effects of C/EBPα overexpression on alveolar epithelial type II cell proliferation, apoptosis and surfactant protein-C expression after exposure to hyperoxia
Source: BMC Pulm Med. 2019 Aug 6;19:142. doi: 10.1186/s12890-019-0911-x (PMC6683353; doi:10.1186/s12890-019-0911-x)
Supplement: Supplementary file 1 — Figure S1. Uncropped Western blots for Fig. 1. Figure S2. Overexpression of C/EBPα promotes cell proliferation after exposure to hyperoxia. Figure S3. Overexpression of C/EBPα decreases cells apoptosis and necrosis after exposure to hyperoxia. Figure S4. The part of original data for Fig. 2. Figure S5. The part of original data for Fig. 3. (PDF 823 kb) [file 12890_2019_911_MOESM1_ESM.pdf]

Effects of C/EBPα overexpression on alveolar epithelial type II cell proliferation, apoptosis and surfactant protein-C expression after exposure to hyperoxia

Hongyan Lu[1]*, Xiaoqing Chen[2]*, Yanmin Lu[1], Haitao Zhu[1], Wei Tang[1], Qiuxia Wang[1]

[1]Department of Pediatrics, Affiliated Hospital of Jiangsu University, Zhenjiang 212000, China

[2]Department of Pediatrics, the First Affiliated Hospital of Nanjing Medical University, Nanjing, Jiangsu, China

*These authors contributed equally to this work.

Corresponding author:

Hongyan Lu

Department of Pediatrics, Affiliated Hospital of Jiangsu University, Zhenjiang 212000, China

Tel.: +86-0511-8508-2260

E-mail: lhy5154@163.com

Supplementary information

Supplementary Materials:

Figure S1 Uncropped Western blots for Figure 1.

Figure S2 Overexpression of C/EBPα promotes cell proliferation after exposure to hyperoxia.

Figure S3 Overexpression of C/EBPα decreases cells apoptosis and necrosis after exposure to hyperoxia.

Figure S4 The part of original data for Figure 2.

Figure S5 The part of original data for Figure 3.

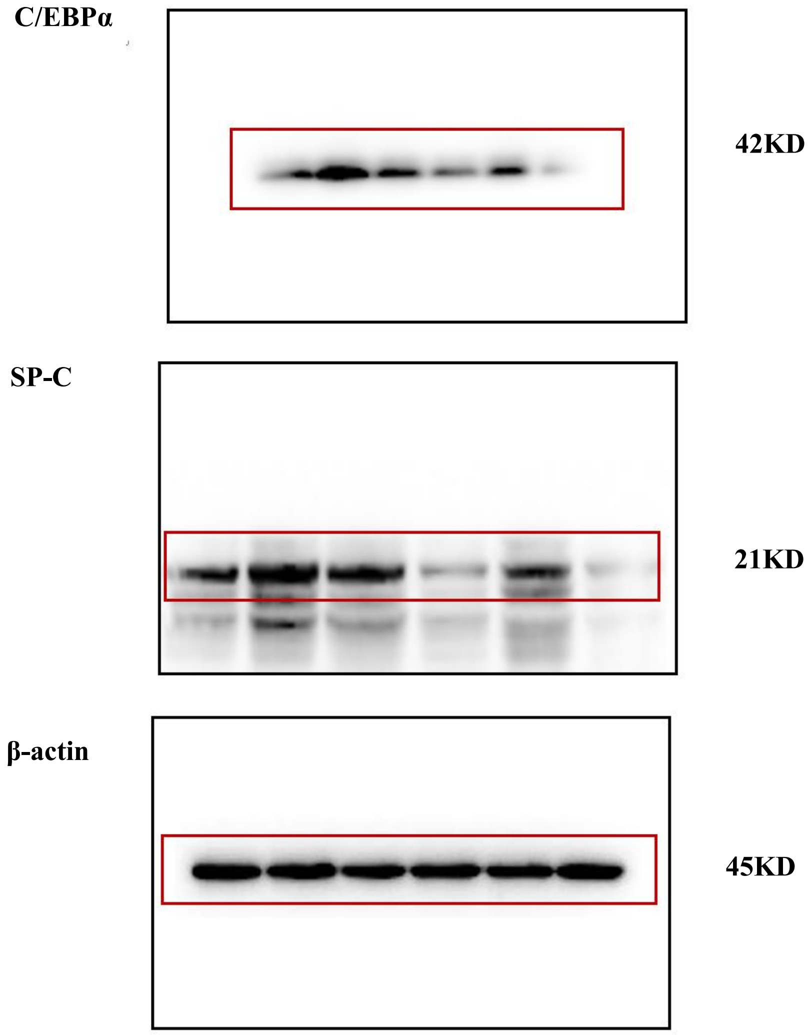

Figure S1 Uncropped Western blots for Figure 1b.

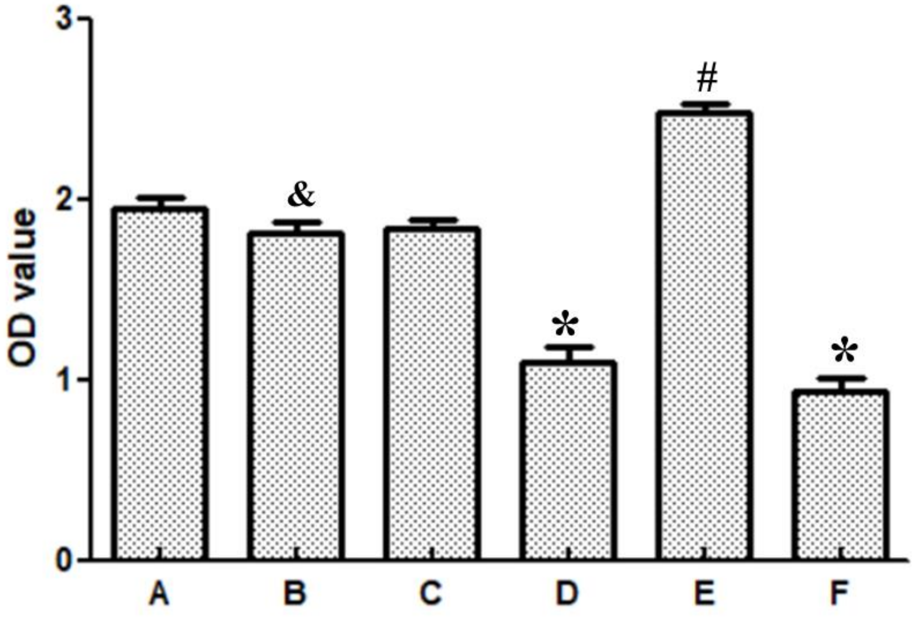

Figure S2. Overexpression of C/EBPα promotes cell proliferation after exposure to hyperoxia. Cell proliferation was analyzed using CCK-8 assay following transfection of C/EBPα after 48 h. Data are presented as the mean ± SD from ten independent experiments (n=10); *P<0.05 vs. AG; $^{\#}$P<0.05 vs. HG or H+E; $^{\&}$P>0.05 vs. AG or A+E. A: AG; B: A+C group; C: A+E group; D: HG; E: H+C group; F: H+E group.

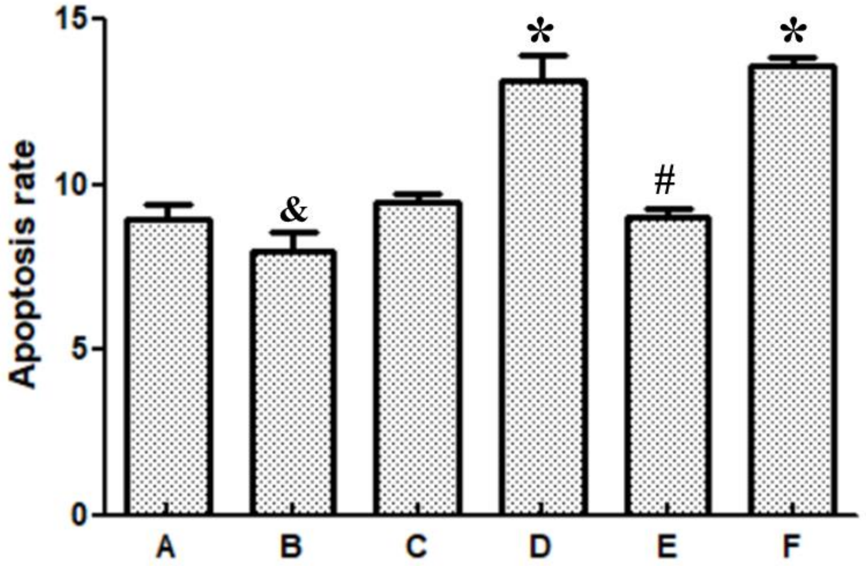

Figure S3. Overexpression of C/EBPα decreases cells apoptosis and

necrosis after exposure to hyperoxia. Apoptosis rate of cells was analyzed by flow cytometry in each group. Values are mean ± SD from ten independent experiments (n=10); *P<0.05 vs. AG; #P<0.05 vs. HG or H+E; &P>0.05 vs. AG or A+E. A: AG; B: A+C group; C: A+E group; D: HG; E: H+C group; F: H+E group.

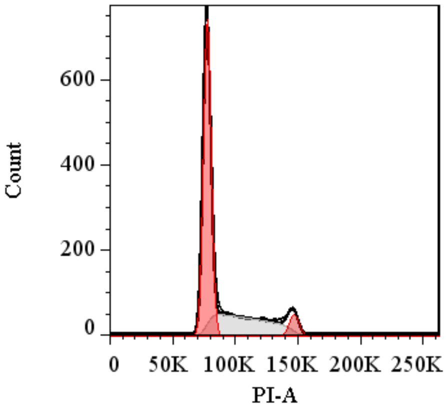

Specimen_001_1_001.fcs
Cell Cycle
Dean-Jett-Fox
RMS = 3.28
Freq. G1 = 64.03
Freq. S = 31.27
Freq. G2 = 5.71
G1 Mean = 77568.11
G2 Mean = 146524.12
G1 cv = 5.62
G2 cv = 3.87
Freq. sub-G1 = -1.77
Freq. super-G2 = -0.88

8731

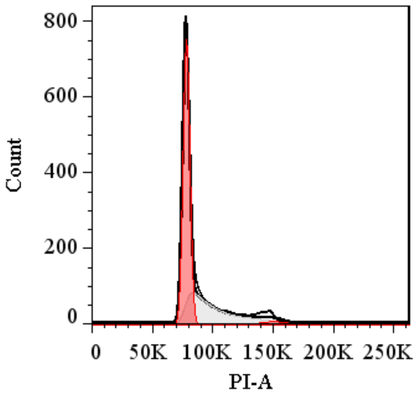

Specimen_001_2_002.fcs
Cell Cycle
Dean-Jett-Fox
RMS = 1.99
Freq. G1 = 61.98
Freq. S = 35.67
Freq. G2 = 2.85
G1 Mean = 78035.33
G2 Mean = 150019.03
G1 cv = 5.05
G2 cv = 10.43
Freq. sub-G1 = -1.99
Freq. super-G2 = -2.38

8290

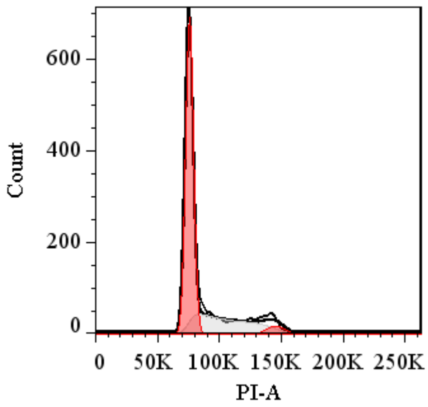

Specimen_001_3_003.fcs
Cell Cycle
Dean-Jett-Fox
RMS = 1.88
Freq. G1 = 68.61
Freq. S = 28.19
Freq. G2 = 4.17
G1 Mean = 75690.77
G2 Mean = 144902.24
G1 cv = 5.97
G2 cv = 7.3
Freq. sub-G1 = -1.29
Freq. super-G2 = -2.09

7901

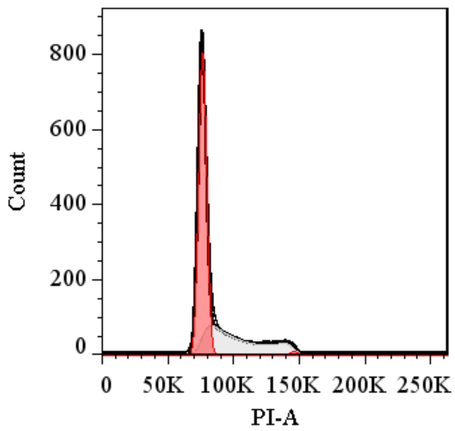

Specimen_001_4_004.fcs
Cell Cycle
Dean-Jett-Fox
RMS = 1.94
Freq. G1 = 64.63
Freq. S = 34.3
Freq. G2 = 0.89
G1 Mean = 76258.2
G2 Mean = 145405.23
G1 cv = 5.54
G2 cv = 2.94
Freq. sub-G1 = -1.17
Freq. super-G2 = -0.73

9211

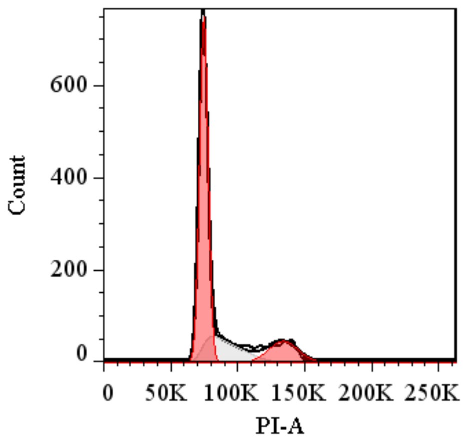

Specimen_001_5_005.fcs
Cell Cycle
Dean-Jett-Fox
RMS = 2.2
Freq. G1 = 67.54
Freq. S = 20.15
Freq. G2 = 14.55
G1 Mean = 74538.88
G2 Mean = 133708.89
G1 cv = 6.04
G2 cv = 11.73
Freq. sub-G1 = -2.12
Freq. super-G2 = -1.62

8635

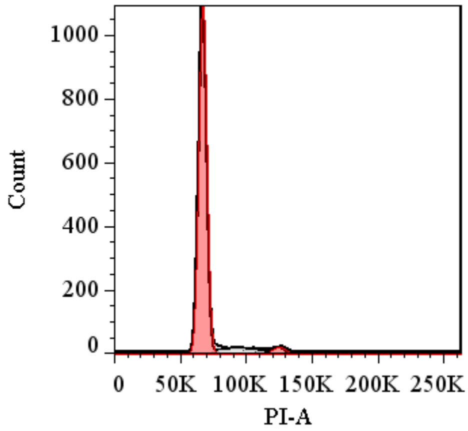

Specimen_001_7_007.fcs
Cell Cycle
Dean-Jett-Fox
RMS = 1.66
Freq. G1 = 91.09
Freq. S = 5.95
Freq. G2 = 3
G1 Mean = 67047.61
G2 Mean = 124783.02
G1 cv = 6.27
G2 cv = 5.62
Freq. sub-G1 = 0.09
Freq. super-G2 = -0.14

9009

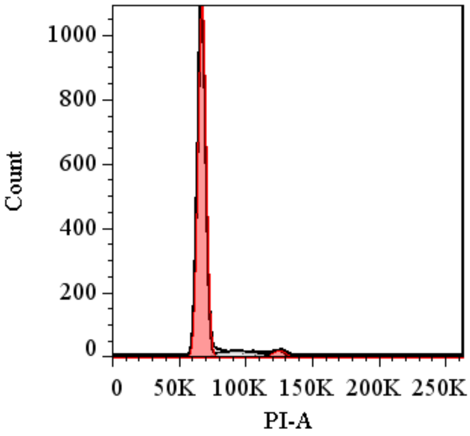

Specimen_001_7_007.fcs
Cell Cycle
Dean-Jett-Fox
RMS = 1.66
Freq. G1 = 91.09
Freq. S = 5.95
Freq. G2 = 3
G1 Mean = 67047.61
G2 Mean = 124783.02
G1 cv = 6.27
G2 cv = 5.62
Freq. sub-G1 = 0.09
Freq. super-G2 = -0.14

9009

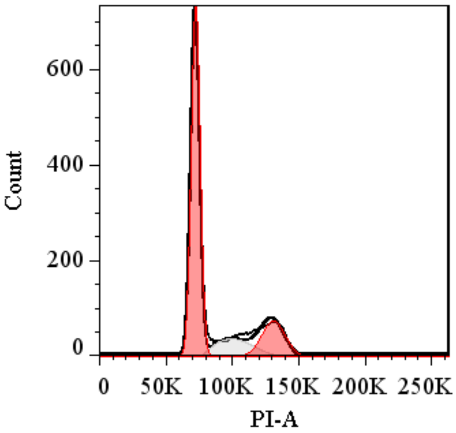

Specimen_001_8_008.fcs
Cell Cycle
Dean-Jett-Fox
RMS = 2.05
Freq. G1 = 68.94
Freq. S = 13.72
Freq. G2 = 18.24
G1 Mean = 72174.83
G2 Mean = 130300.12
G1 cv = 6.5
G2 cv = 9.47
Freq. sub-G1 = 0.25
Freq. super-G2 = -1.2

8749

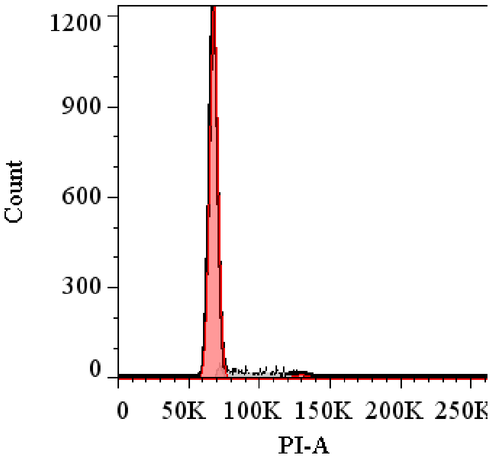

Specimen_001_9_009.fcs
Cell Cycle
Watson
RMS = 3.06
Freq. G1 = 92.29
Freq. S = 6.41
Freq. G2 = 2.01
G1 Mean = 67645.4
G2 Mean = 129243.22
G1 cv = 6.06
G2 cv = 6.96
Freq. sub-G1 = 0.35
Freq. super-G2 = -0.1

9700

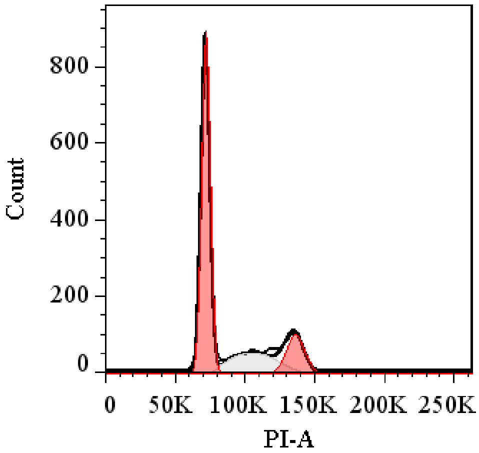

Specimen_001_10_010.fcs
Cell Cycle
Dean-Jett-Fox
RMS = 1.69
Freq. G1 = 64.35
Freq. S = 20.16
Freq. G2 = 15.13
G1 Mean = 71850.73
G2 Mean = 135678.09
G1 cv = 6.1
G2 cv = 6.8
Freq. sub-G1 = 0.61
Freq. super-G2 = -0.73

10479

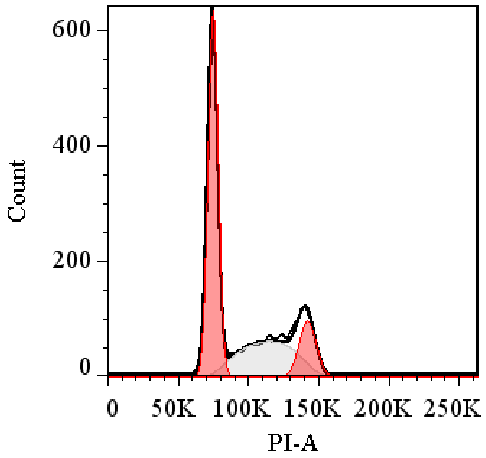

Specimen_001_11_011.fcs
Cell Cycle
Dean-Jett-Fox
RMS = 1.66
Freq. G1 = 55.46
Freq. S = 30.21
Freq. G2 = 14.04
G1 Mean = 74561.79
G2 Mean = 141496.77
G1 cv = 6.86
G2 cv = 6.01
Freq. sub-G1 = 0.23
Freq. super-G2 = -1.59

10129

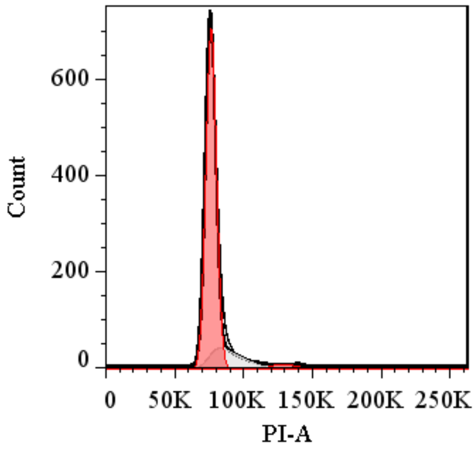

Specimen_001_9_009.fcs
Cell Cycle
Dean-Jett-Fox
RMS = 2.02
Freq. G1 = 82.93
Freq. S = 14.51
Freq. G2 = 2.78
G1 Mean = 76229.52
G2 Mean = 128915.29
G1 cv = 7.02
G2 cv = 9.79
Freq. sub-G1 = -1.7
Freq. super-G2 = -0.03

7915

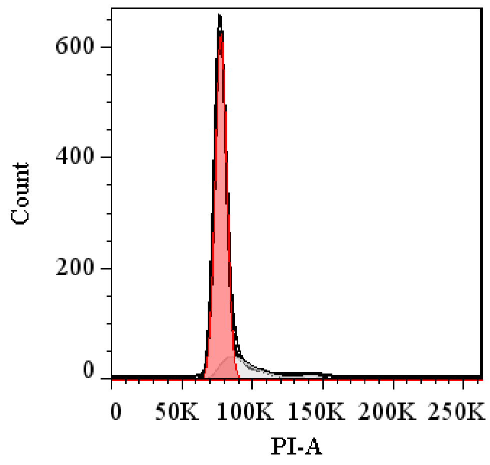

Specimen_001_8_008.fcs
Cell Cycle
Dean-Jett-Fox
RMS = 1.52
Freq. G1 = 81.24
Freq. S = 17.75
Freq. G2 = 0.28
G1 Mean = 77480.68
G2 Mean = 147157.25
G1 cv = 7.38
G2 cv = 2.87
Freq. sub-G1 = -1.08
Freq. super-G2 = 0.31

7617

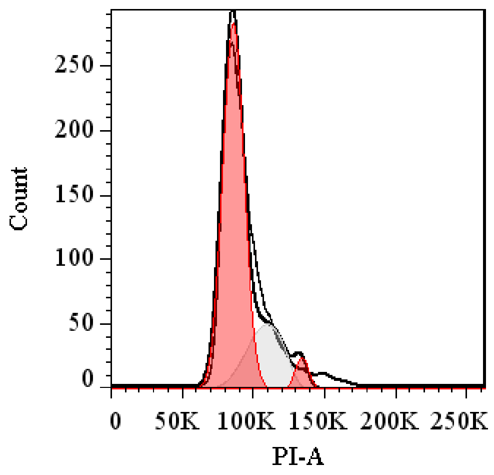

Specimen_001_7_007.fcs
Cell Cycle
Dean-Jett-Fox
RMS = 2.34
Freq. G1 = 73.41
Freq. S = 21.89
Freq. G2 = 3.86
G1 Mean = 85670.99
G2 Mean = 133703.71
G1 cv = 12.31
G2 cv = 4.86
Freq. sub-G1 = -2.18
Freq. super-G2 = 2.12

7043

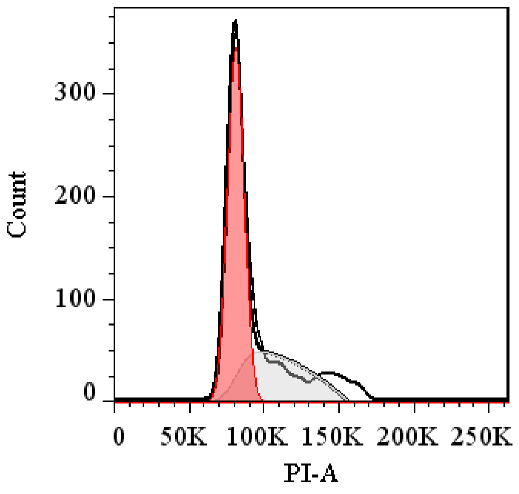

Specimen_001_1_001.fcs
Cell Cycle
Dean-Jett-Fox
RMS = 2.16
Freq. G1 = 62.96
Freq. S = 34.83
Freq. G2 = 0
G1 Mean = 80814.93
G2 Mean = 158543.03
G1 cv = 9.71
G2 cv = 0
Freq. sub-G1 = -1.84
Freq. super-G2 = 2.13

7428

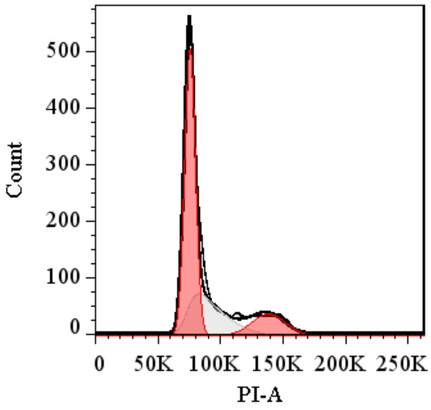

Specimen_001_8_008.fcs
Cell Cycle
Dean-Jett-Fox
RMS = 2.67
Freq. G1 = 58.81
Freq. S = 27.13
Freq. G2 = 13.95
G1 Mean = 75396.58
G2 Mean = 138000.13
G1 cv = 8.05
G2 cv = 13.83
Freq. sub-G1 = -2.75
Freq. super-G2 = -0.45

9050

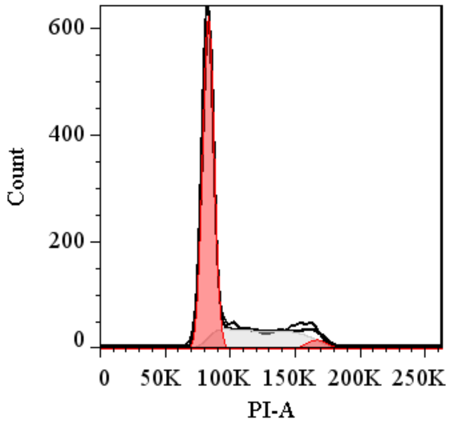

Specimen_001_7_007.fcs
Cell Cycle
Dean-Jett-Fox
RMS = 2.6
Freq. G1 = 67.29
Freq. S = 28.16
Freq. G2 = 3.13
G1 Mean = 82902.17
G2 Mean = 166150.12
G1 cv = 7.48
G2 cv = 6.62
Freq. sub-G1 = 0.21
Freq. super-G2 = -1.23

9939

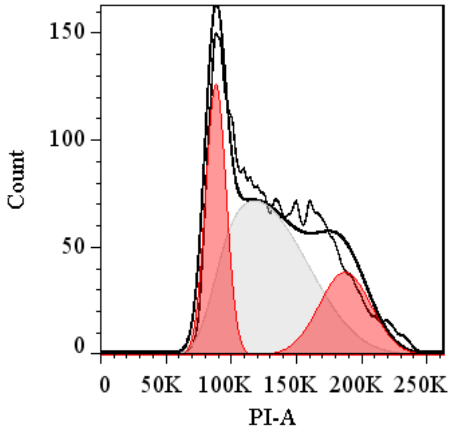

Specimen_001_6_006.fcs
Cell Cycle
Dean-Jett-Fox
RMS = 2.26
Freq. G1 = 24.49
Freq. S = 56.11
Freq. G2 = 18.87
G1 Mean = 87947.44
G2 Mean = 186365.42
G1 cv = 12.53
G2 cv = 14.97
Freq. sub-G1 = -3.39
Freq. super-G2 = -1.95

9830

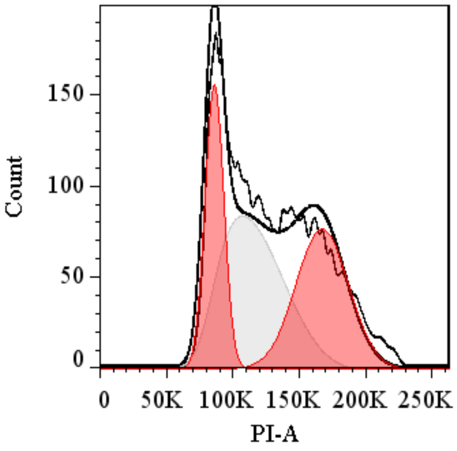

Specimen_001_6-1_010.fcs
Cell Cycle
Dean-Jett-Fox
RMS = 2.71
Freq. G1 = 23.44
Freq. S = 42.51
Freq. G2 = 32.31
G1 Mean = 85812.73
G2 Mean = 166617.52
G1 cv = 11.57
G2 cv = 16.72
Freq. sub-G1 = -3.38
Freq. super-G2 = 0.76

11415

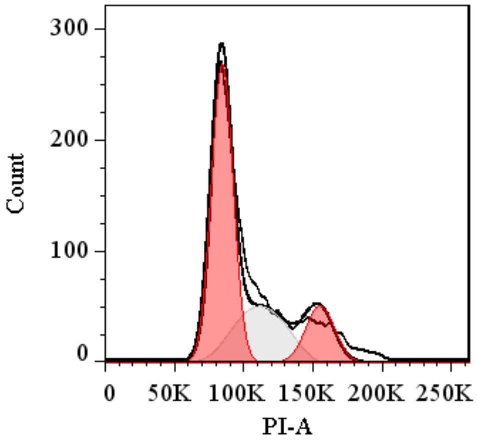

Specimen_001_5_005.fcs
Cell Cycle
Dean-Jett-Fox
RMS = 2.3
Freq. G1 = 57.92
Freq. S = 26.03
Freq. G2 = 14.81
G1 Mean = 84220.76
G2 Mean = 154600.74
G1 cv = 13.11
G2 cv = 9.51
Freq. sub-G1 = -1.76
Freq. super-G2 = 1.91

8858

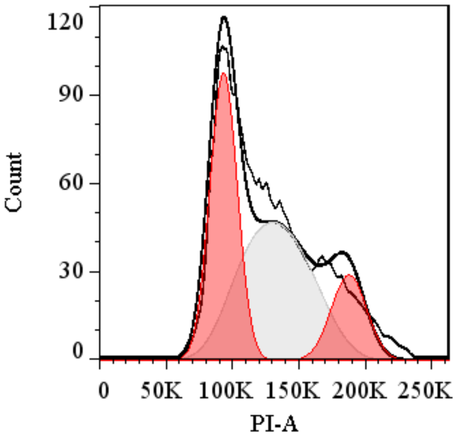

Specimen_001_4_004.fcs
Cell Cycle
Dean-Jett-Fox
RMS = 1.63
Freq. G1 = 36.97
Freq. S = 47.99
Freq. G2 = 14.13
G1 Mean = 92930.39
G2 Mean = 186964.95
G1 cv = 15.71
G2 cv = 10.06
Freq. sub-G1 = -2.81
Freq. super-G2 = 0.04

6672

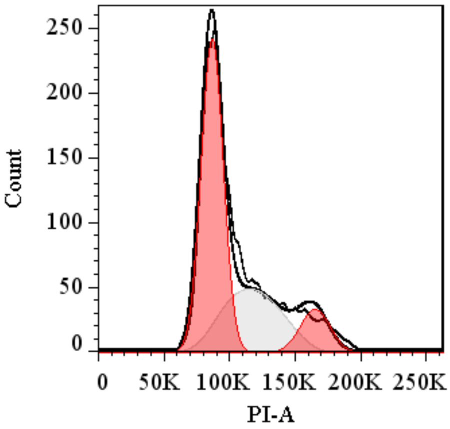

Specimen_001_3_003.fcs
Cell Cycle
Dean-Jett-Fox
RMS = 1.83
Freq. G1 = 57.94
Freq. S = 31.74
Freq. G2 = 10.81
G1 Mean = 86401.62
G2 Mean = 164565.05
G1 cv = 13.77
G2 cv = 9.78
Freq. sub-G1 = -2.64
Freq. super-G2 = -0.29

8593

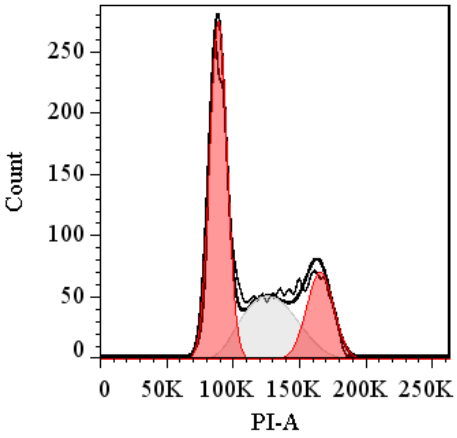

Specimen_001_2_002.fcs
Cell Cycle
Dean-Jett-Fox
RMS = 2.24
Freq. G1 = 51.42
Freq. S = 29.77
Freq. G2 = 20.24
G1 Mean = 88614.03
G2 Mean = 164816.79
G1 cv = 10.43
G2 cv = 8.6
Freq. sub-G1 = -0.75
Freq. super-G2 = -1.88

8537

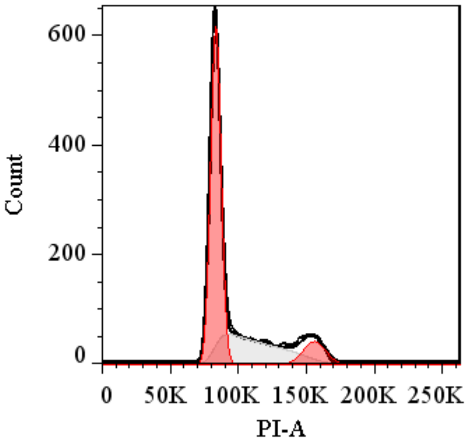

Specimen_001_1_001.fcs
Cell Cycle
Dean-Jett-Fox
RMS = 1.69
Freq. G1 = 61.45
Freq. S = 29.82
Freq. G2 = 9.16
G1 Mean = 83200.27
G2 Mean = 155326.97
G1 cv = 6.46
G2 cv = 7.58
Freq. sub-G1 = -1.55
Freq. super-G2 = -1.44

9356

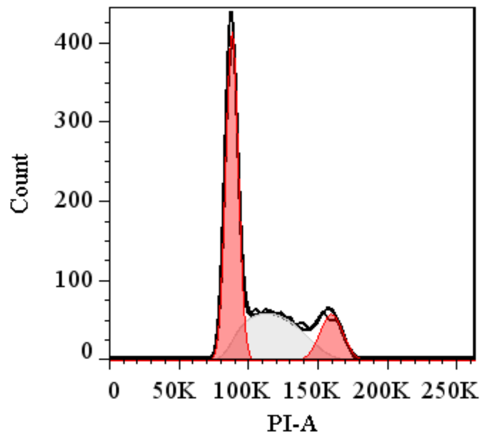

Specimen_001_2_002.fcs
Cell Cycle
Dean-Jett-Fox
RMS = 1.48
Freq. G1 = 52.39
Freq. S = 35.16
Freq. G2 = 13.56
G1 Mean = 87819.2
G2 Mean = 158987.09
G1 cv = 7.2
G2 cv = 7.34
Freq. sub-G1 = -1.74
Freq. super-G2 = -1.15

8621

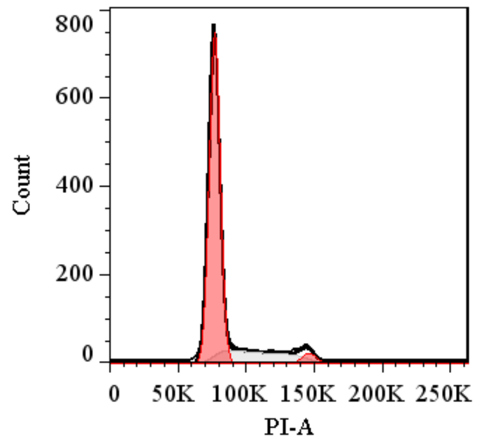

Specimen_001_3_003.fcs
Cell Cycle
Dean-Jett-Fox
RMS = 1.68
Freq. G1 = 78.18
Freq. S = 17.85
Freq. G2 = 3.13
G1 Mean = 76763.59
G2 Mean = 145708.77
G1 cv = 7.41
G2 cv = 5.05
Freq. sub-G1 = 0.04
Freq. super-G2 = -0.6

9408

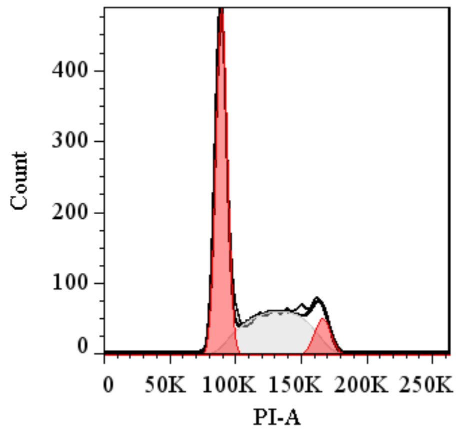

Specimen_001_4_004.fcs
Cell Cycle
Dean-Jett-Fox
RMS = 1.46
Freq. G1 = 53.39
Freq. S = 38.55
Freq. G2 = 8.1
G1 Mean = 89066.93
G2 Mean = 165490.98
G1 cv = 6.81
G2 cv = 5.32
Freq. sub-G1 = -0.54
Freq. super-G2 = -1.56

9554

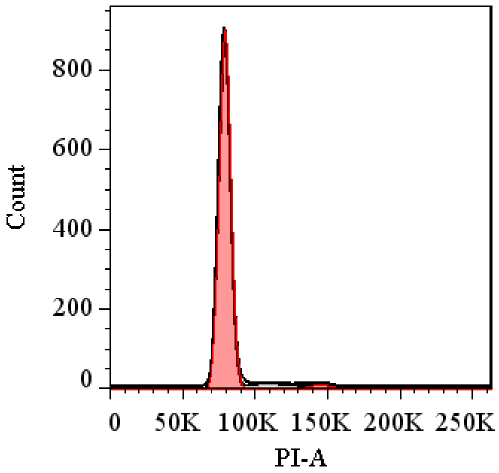

Specimen_001_5_005.fcs
Cell Cycle
Dean-Jett-Fox
RMS = 1.59
Freq. G1 = 93.34
Freq. S = 4.05
Freq. G2 = 2.44
G1 Mean = 79324.05
G2 Mean = 144881.71
G1 cv = 7.09
G2 cv = 8.25
Freq. sub-G1 = 0.48
Freq. super-G2 = -0.36

9430

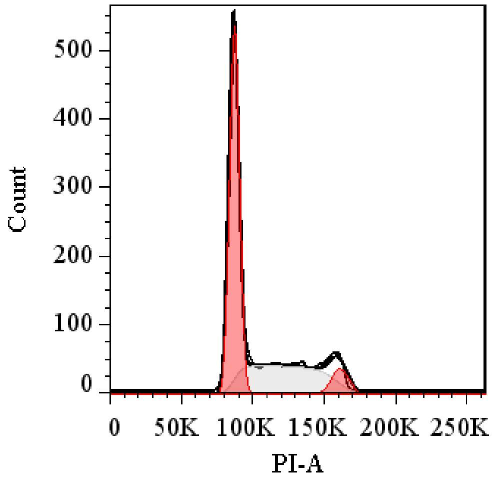

Specimen_001_6_006.fcs
Cell Cycle
Dean-Jett-Fox
RMS = 1.62
Freq. G1 = 57.73
Freq. S = 35.65
Freq. G2 = 7.05
G1 Mean = 87045.58
G2 Mean = 160512.1
G1 cv = 5.62
G2 cv = 5.43
Freq. sub-G1 = -0.53
Freq. super-G2 = -2.33

7831

G1
G2
S Phase
Sum
Statistic

Figure S4 The part of original data for Figure 2.

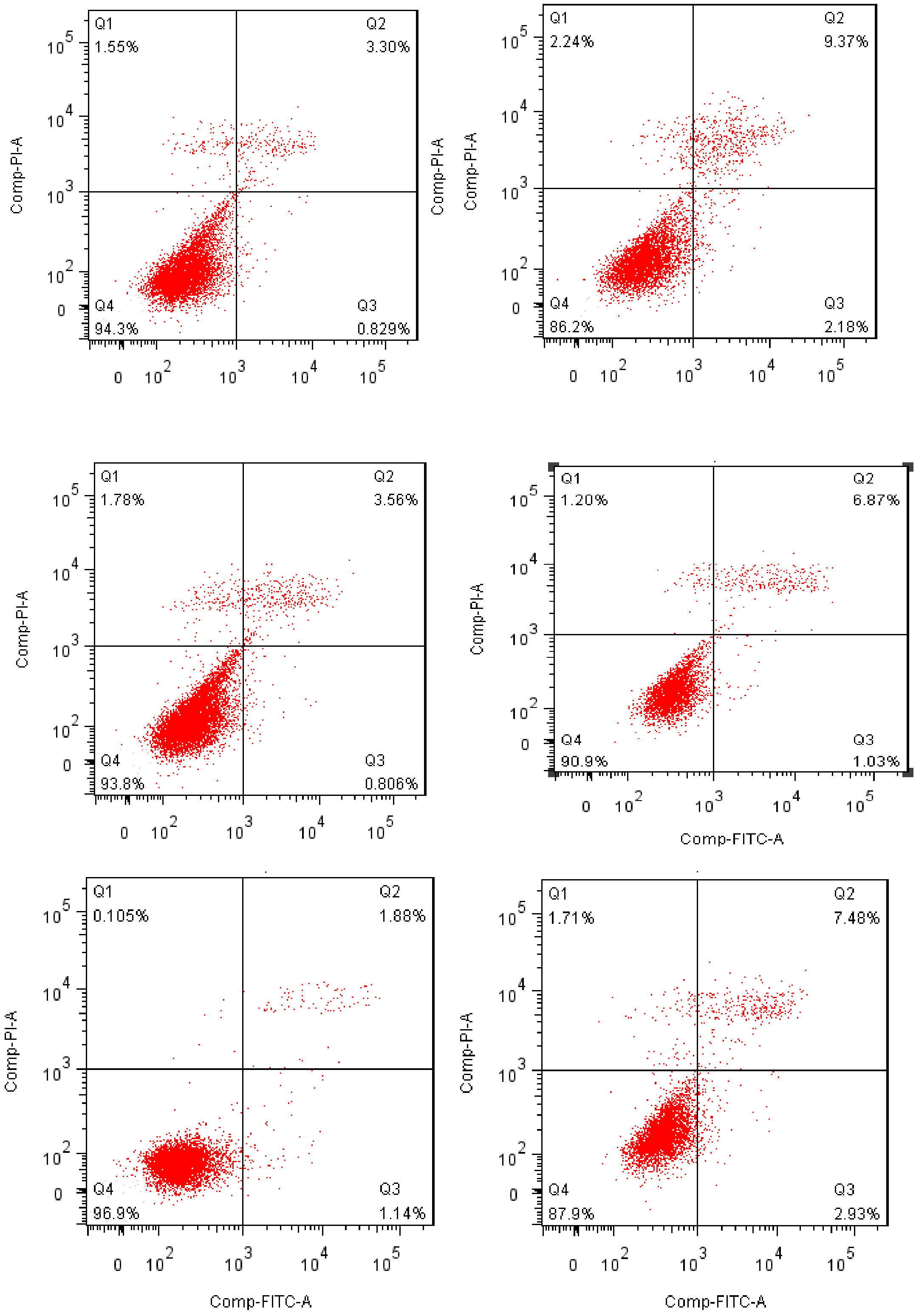
Q1
1.55%
Q2
3.30%
Q4
94.3%
Q3
0.829%
Comp-PI-A
Q1
2.24%
Q2
9.37%
Q4
86.2%
Q3
2.18%
Comp-PI-A
Q1
1.78%
Q2
3.56%
Q4
93.8%
Q3
0.806%
Comp-PI-A
Q1
1.20%
Q2
6.87%
Q4
90.9%
Q3
1.03%
Comp-PI-A
Comp-FITC-A
Q1
0.105%
Q2
1.88%
Q4
96.9%
Q3
1.14%
Comp-PI-A
Comp-FITC-A
Q1
1.71%
Q2
7.48%
Q4
87.9%
Q3
2.93%
Comp-PI-A
Comp-FITC-A

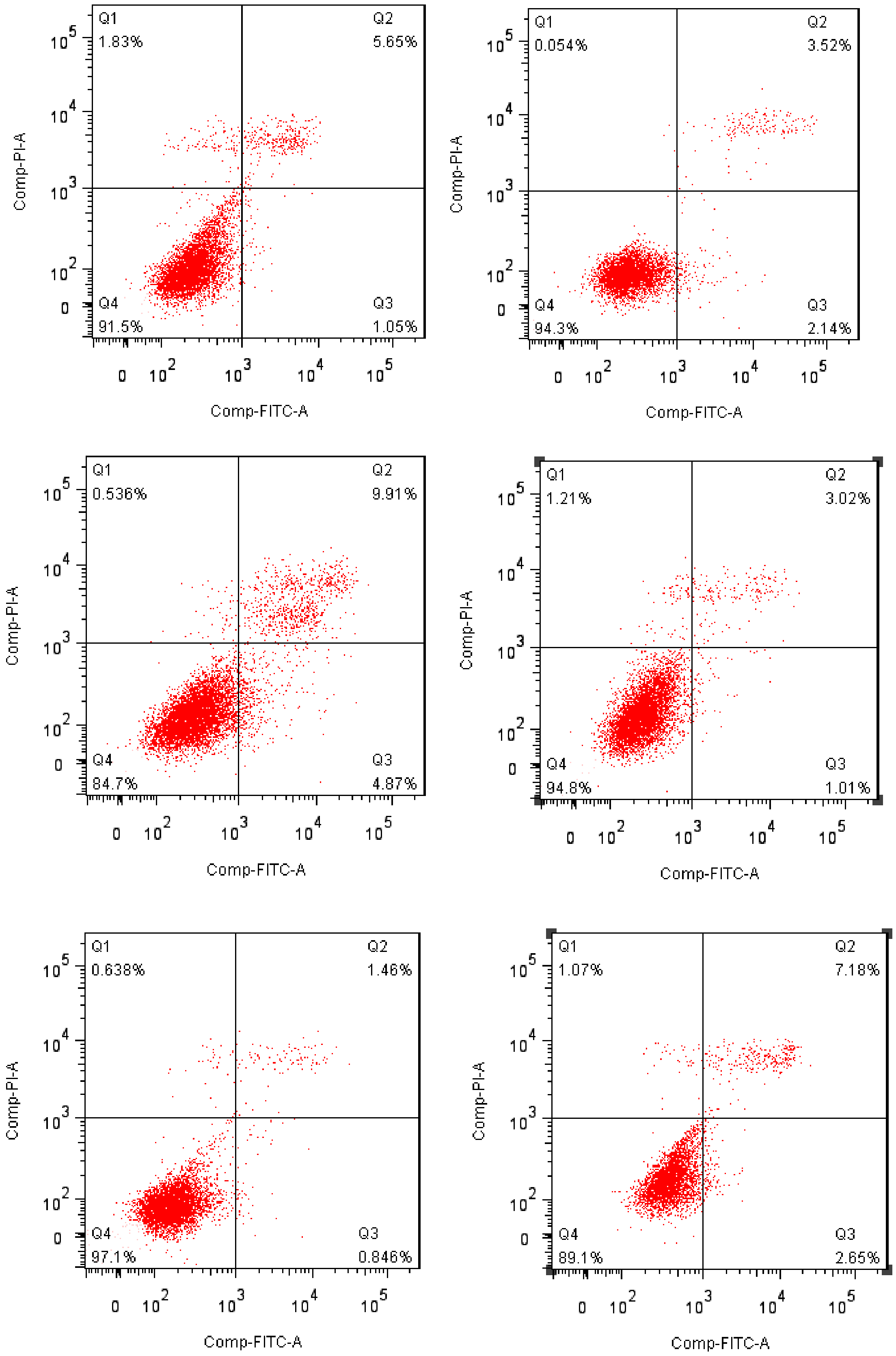

Q1
1.83%
Q2
5.65%
Q4
91.5%
Q3
1.05%
Comp-PI-A
Comp-FITC-A
Q1
0.054%
Q2
3.52%
Q4
94.3%
Q3
2.14%
Comp-PI-A
Comp-FITC-A
Q1
0.536%
Q2
9.91%
Q4
84.7%
Q3
4.87%
Comp-PI-A
Comp-FITC-A
Q1
1.21%
Q2
3.02%
Q4
94.8%
Q3
1.01%
Comp-PI-A
Comp-FITC-A
Q1
0.638%
Q2
1.46%
Q4
97.1%
Q3
0.846%
Comp-PI-A
Comp-FITC-A
Q1
1.07%
Q2
7.18%
Q4
89.1%
Q3
2.65%
Comp-PI-A
Comp-FITC-A

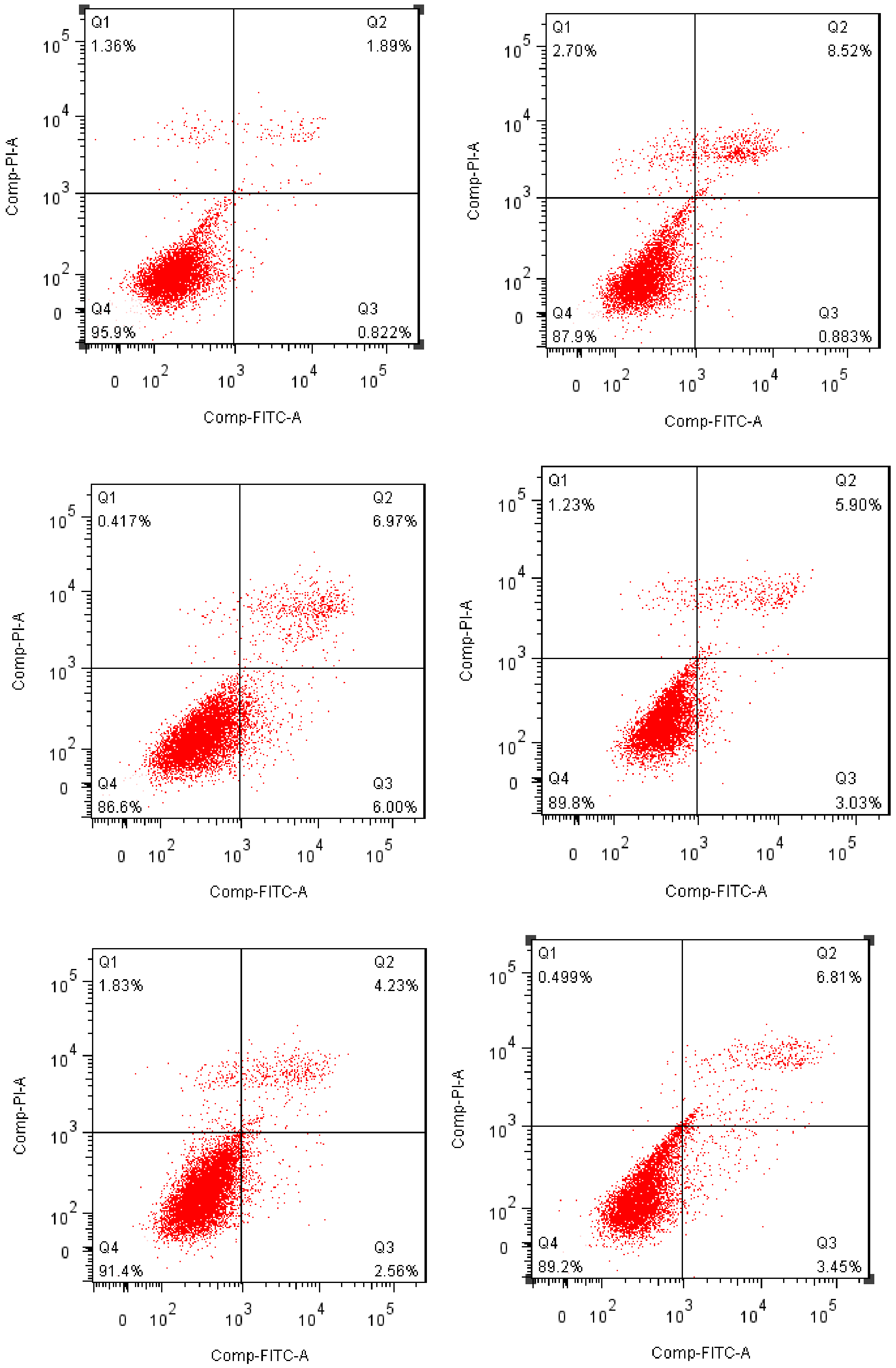
Q1 1.36%
Q2 1.89%
Q4 95.9%
Q3 0.822%
Comp-PI-A
Comp-FITC-A
Q1 2.70%
Q2 8.52%
Q4 87.9%
Q3 0.883%
Comp-PI-A
Comp-FITC-A
Q1 0.417%
Q2 6.97%
Q4 86.6%
Q3 6.00%
Comp-PI-A
Comp-FITC-A
Q1 1.23%
Q2 5.90%
Q4 89.8%
Q3 3.03%
Comp-PI-A
Comp-FITC-A
Q1 1.83%
Q2 4.23%
Q4 91.4%
Q3 2.56%
Comp-PI-A
Comp-FITC-A
Q1 0.499%
Q2 6.81%
Q4 89.2%
Q3 3.45%
Comp-PI-A
Comp-FITC-A

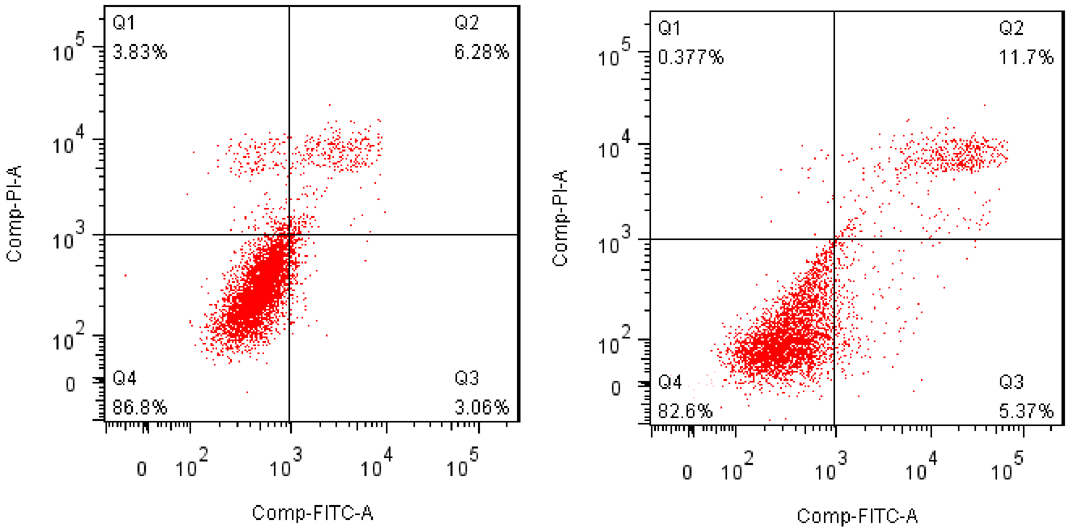

Figure S5 The part of original data for Figure 3.
